# Lateral Habenula Regulates Cardiovascular Autonomic Responses *via* the Serotonergic System in Rats

**DOI:** 10.3389/fnins.2021.655617

**Published:** 2021-03-29

**Authors:** Tri Huu Doan, Yuma Sato, Masayuki Matsumoto, Tadachika Koganezawa

**Affiliations:** ^1^Department of Physiology, Division of Biomedical Science, Faculty of Medicine, University of Tsukuba, Tsukuba, Japan; ^2^Doctoral Program in Biomedical Sciences, Graduate School of Comprehensive Human Sciences, University of Tsukuba, Tsukuba, Japan; ^3^Center for Advanced Training in Clinical Simulation, University of Medicine and Pharmacy at Ho Chi Minh City, Ho Chi Minh City, Vietnam; ^4^Master’s Program in Medical Sciences, Graduate School of Comprehensive Human Sciences, University of Tsukuba, Tsukuba, Japan; ^5^Department of Cognitive and Behavioral Neuroscience, Division of Biomedical Science, Faculty of Medicine, University of Tsukuba, Tsukuba, Japan; ^6^Transborder Medical Research Center, University of Tsukuba, Tsukuba, Japan

**Keywords:** autonomic nervous system, sympathetic nervous system, parasympathetic nervous system, stress, serotonergic system, cardiovascular

## Abstract

The lateral habenula (LHb) plays essential roles in behavioral responses to stressful events. Stress is tightly linked to autonomic responses such as cardiovascular responses, yet how the LHb regulates these responses is not well understood. To address this issue, we electrically stimulated the LHb in rats, measured its effects on heart rate (HR) and mean arterial pressure (MAP), and investigated the neural circuits that mediate these LHb-induced cardiovascular responses *via* the autonomic nervous system. We observed that stimulation of the LHb induced bradycardia and pressor responses, whereas stimulation of the adjacent areas changed neither the HR nor the MAP. Bilateral vagotomy and administration of a muscarinic receptor antagonist suppressed the LHb stimulation effect on the HR but not on the MAP, whereas administration of a β-adrenoceptor antagonist partly attenuated the effect on the MAP but not on the HR. Thus, the LHb-induced cardiovascular responses of the HR and the MAP were likely caused by activations of the cardiac parasympathetic nerves and the cardiovascular sympathetic nerves, respectively. Furthermore, administration of a non-selective 5-HT receptor antagonist significantly attenuated the LHb stimulation effects on both the MAP and the HR. A 5-HT_2_ receptor antagonist also attenuated the LHb stimulation effects. A low dose of a 5-HT_1A_ receptor antagonist enhanced the LHb stimulation effects, but a high dose of the drug attenuated them. 5-HT_1B_ and 5-HT_1D_ receptor antagonists as well as a 5-HT_7_ receptor antagonist did not affect the LHb stimulation effects. Taken together, our findings suggest that the LHb regulates autonomic cardiovascular responses at least partly through the serotonergic system, particularly *via* the 5-HT_1A_ and 5-HT_2_ receptors.

## Introduction

The autonomic nervous system, especially the autonomic cardiovascular system, plays critical roles to ensure the survival of animals, including humans, when they face perceived threats in the environment. The autonomic nervous system consists of two components: the sympathetic nervous system and the parasympathetic nervous system. The sympathetic nervous system accelerates the cardiovascular system and leads to pressor responses by increasing heart rate (HR) and contractility as well as by causing constriction of the peripheral arterioles. In contrast, the parasympathetic nervous system causes opposite effects, which include reduction of HR and contractility, and consequently, decreases cardiac output and hence blood pressure. These systems are mainly regulated by two mechanisms: (1) a reflex originating from peripheral sensory neuron impulses and (2) a central mechanism, or “central command,” which adapts the blood circulation *via* modulation from higher centers in the brain (Dampney, 2016).

An essential factor that elicits the central command is acute psychological stimuli, such as smell and sight of predators, which indicate threatening and stressful occasions and need to be responded to as fast as possible. The brain analyzes these acute psychological stimuli and chooses appropriate behavioral responses to them depending on the environmental demands. These responses are called coping strategies and are divided into “active” and “passive” strategies (Bandler et al., 2000). Active coping strategies, such as fight or flight, are suitable if the acute psychological stimuli are escapable, and these strategies are commonly accompanied by facilitation of sympathetic outflows with increasing HR and systemic arterial pressure (Hilton, 1982). In contrast, passive coping strategies, which include immobilization or freezing, are activated when the stimuli are inescapable, and these strategies are commonly accompanied by activation of the sympathetic and parasympathetic nervous systems along with causing bradycardia and elevation of systemic blood pressure (Schenberg et al., 1993; Schadt and Hasser, 1998; Carrive, 2006).

A major candidate for the regulation of these coping strategies in the brain is the lateral habenula (LHb). The LHb is a part of the habenular complex located at the epithalamus and is considered as a station to connect the limbic system and basal ganglia *via* the dopaminergic system (the substantia nigra pars compacta and ventral tegmental area) and the serotonergic system (the dorsal and median raphé nuclei) (Herkenham and Nauta, 1977; Hikosaka et al., 2008; Weissbourd et al., 2014). It has been reported that neurons in the LHb respond to stressful stimuli (Gao et al., 1996; Matsumoto and Hikosaka, 2009; Kawai et al., 2015; Li et al., 2019) and regulate the activities of dopamine neurons (Christoph et al., 1986; Ji and Shepard, 2007; Matsumoto and Hikosaka, 2007; Lecourtier et al., 2008; Jhou et al., 2009) and serotonin neurons (Ferraro et al., 1996; Amat et al., 2001). These LHb signals are known to control behavioral responses to stressful events (Thornton and Bradbury, 1989; Lecourtier et al., 2004; Stamatakis and Stuber, 2012; Mathis et al., 2018). Particularly, it has been shown that the LHb regulates fighting behavior (i.e., an active coping strategy) as well as freezing (i.e., a passive coping strategy) (Agetsuma et al., 2010; Chou et al., 2016; Nakajo et al., 2020), which are accompanied by the cardiovascular responses described above. However, despite abundant studies on the roles of the LHb in stress-induced behavioral responses, how it regulates autonomic responses, including cardiovascular responses, is unclear.

To address this issue, we here electrically stimulated the LHb in rats, measured its effects on HR and mean arterial pressure (MAP), and investigated the neural circuits that mediate these LHb-induced cardiovascular responses *via* the autonomic nervous system. We found that activation of the LHb induces bradycardia and a pressor response *via* the cardiac parasympathetic nerves and the cardiovascular sympathetic nerves, respectively. Pharmacologic experiments suggested that these LHb-induced cardiovascular responses are mediated at least partly through the serotonergic system. Our findings extend the current knowledge on the function of the LHb by highlighting its role in autonomic regulation and the underlying neural circuit.

## Materials and Methods

### Ethical Approval

The animal study was reviewed and approved by the Animal Experimental Committee of the University of Tsukuba (permission numbers: 17-119, 18-027, 19-016, and 20-019).

### Animal Preparation

All experiments were conducted on male Wistar rats (Japan SLC, Inc.^1^) weighing 260–330 g at the time of the experiments. All the rats were maintained under standard laboratory conditions with a 12-h light/12-h dark normal cycle (lights on at 7 AM) at 25°C and had free access to water and food. On the day of the experiment, anesthesia was initially induced with isoflurane (Fujifilm Wako Pure Chemical Corporation), and the anesthetized state was maintained with urethane (1–1.25 g/kg body weight, i.p.; Tokyo Chemical Industry Co., Ltd.). The dose of the anesthetic agent was based on those of previous studies (Martin et al., 2006; Drew et al., 2011), and the depth of anesthesia was confirmed by a negative reflex on the paw-pinch test. A heparinized saline-filled polyethylene catheter (SP31; Natsume Seisakusho Co., Ltd.) was inserted into the left femoral artery and connected to a carrier amplifier (AP-621G; Nihon Kohden) to record the MAP. The HR was calculated from lead-I electrocardiography, which was amplified by the use of a bioelectrical amplifier (AB-651J; Nihon Kohden). The right femoral vein was cannulated with a saline-filled polyethylene catheter (SP31; Natsume Seisakusho Co., Ltd.) to apply the drugs intravenously. In the vagotomy experiments, described below, before the rats were positioned in a stereotaxic apparatus in the prone position, the bilateral vagus nerves were identified at the neck and threads were detained under the nerves as markers in the supine position.

### Electrical Stimulation of the LHb

The rats were positioned in a stereotaxic apparatus and warmed with a heating pad. Burr-hole craniotomy (3 × 4 mm) was performed to stimulate the LHb at the left parietal bone, and the center of the hole was 3.5 mm caudal to the bregma. The position of the left LHb (3.5–3.8 mm caudal from the bregma, 0.5–0.7 mm lateral from the midline, and 4.5–5.0 mm below the cortical surface) was identified on the basis of the atlas of Paxinos and Watson (1986) and previous reports (Ootsuka and Mohammed, 2015; Ootsuka et al., 2017). A coated electrode (200-μm tip diameter, insulated with polyurethane except for the exposed tip) was inserted vertically into the left LHb, and electrical stimulation of 300-μA intensity, 0.5-ms duration, and 100-Hz frequency was delivered for 10 s (Friedman et al., 2011). Each stimulation was performed at least 60 s apart. The stimulations were repeatable and reversible.

### Mapping of Effective Stimulation Sites Inducing Changes in HR and MAP

We electrically stimulated not only the insides but also the outsides of the LHb to test whether effective stimulation sites, which induced changes in HR and MAP, were centered at the LHb. For each rat (*n* = 13), 3–16 sites inside and 2–30 sites outside the LHb were electrically stimulated. Each stimulation site was at least 0.25 mm apart. The stimulation sites outside the LHb were located mainly in the stria medullaris on the dorsal side, the medial mediodorsal thalamus and the central mediodorsal thalamus nucleus on the ventral side, and the lateral mediodorsal thalamus on the lateral side.

### Blockade of the Sympathetic and Parasympathetic Nerves

Three groups of rats were used to investigate the roles of the sympathetic and parasympathetic nervous systems in the cardiovascular responses to stimulation of the LHb. In the first group of rats (*n* = 5), the cardiac sympathetic nerves to the heart were blocked by systemic administration of a β-adrenoceptor antagonist, propranolol (5 and 10 mg/kg, i.v.; AstraZeneca). In the second group (*n* = 5), the cardiac parasympathetic nerves were blocked by incision of the bilateral vagus nerves at the neck level. In the third group, the cardiac parasympathetic nerves were blocked by systemic administration of a muscarinic receptor antagonist, atropine sulfate (5 mg/kg, i.v.; Fujifilm Wako Pure Chemical Corporation) (Merrick et al., 1979). In each experiment, the same site inside the LHb was electrically stimulated before and after either drug administration or vagotomy.

### Pharmacologic Investigation of the Role of the Serotonergic System in LHb-Induced Cardiovascular Responses

To investigate the role of the serotonergic system in the cardiovascular responses to LHb stimulation, we intravenously applied the following antagonists into five groups of rats: a non-selective 5-HT receptor antagonist, methysergide (1 mg/kg, i.v., *n* = 5; Abcam); a 5-HT_1A_ receptor antagonist, NAD-299 (0.3 and 0.9 mg/kg, i.v., *n* = 6; Tocris Bioscience); a 5-HT_1B_ and 5-HT_1D_ receptor antagonist, GR-127935 (3 and 6 mg/kg, i.v., *n* = 5; Abcam); a 5-HT_2_ receptor antagonist, mianserin (5 mg/kg, i.v., *n* = 5; Tokyo Chemical Industry Co., Ltd.); and a 5-HT_7_ receptor antagonist, SB-269970 (1 and 2 mg/kg, i.v., *n* = 5; Abcam). The dose of each drug was based on those of previous reports (Cavero et al., 1981; Terrón, 1997; Chaouloff et al., 1999; Madjid et al., 2006; Nikiforuk et al., 2013). All the drugs were diluted in saline and applied *via* the femoral venous catheter. Each rat was electrically stimulated at the same site in the LHb before and after administration of each drug. Each animal received only one of the 5-HT receptor antagonists. In case of applying two doses of the drug, each animal was firstly administered the lower dose of the drug and secondly received an additional dose, i.e., the higher dose indicates the total administered dose.

### Histologic Examination

At the end of each experiment, an electric lesion was made by a direct current (100 μA for 10 s) at one of the stimulated sites in the brain. Then, the rat was perfused with 200 ml of saline followed by 200 ml of 10% formalin (Fujifilm Wako Pure Chemical Corporation) through the left ventricle. The brain was removed from the body, immersed in 10% formalin, and stored at 4°C for at least 24 h before 50-μm frozen sections were cut coronally to identify the location of the electrode.

### Data Analysis

All data were digitized by the use of an AD converter (Cambridge Electronic Design Limited) and sampled and analyzed by the use of Spike2 (Cambridge Electronic Design Limited). The data were managed using Excel 2019 (Microsoft) and analyzed using IBM SPSS Statistics 25 (IBM), and graphs were built using Prism 6.0 (GraphPad Software). The HR and MAP values before the LHb stimulation (control) were calculated from the means for 10 s just before the stimulation. When clear responses of HR and MAP were observed, the peak changes were observed in 6–10 s and 3–6 s after starting the stimulation, respectively. Therefore, the responses of HR and MAP were calculated from the differences between the prestimulus control value and the mean value in each period, i.e., 6–10 s after starting the stimulation for HR and 3–6 s for MAP. To analyze the effects of drug administration or vagotomy on the LHb-induced HR and MAP responses, the response magnitudes were expressed as percent changes from the HR and MAP values before the LHb stimulation. All the data were expressed as mean ± SD. To statistically compare two different groups, we used the two-tailed paired or unpaired *t* test. To compare three different groups, we used repeated-measures ANOVA with a *post hoc* Tukey’s test. For the ratio statistics, we used the chi-square (χ^2^) test. Significant *p* values were set at <0.05.

## Results

### Changes in HR and MAP Evoked by Electrical Stimulation of the LHb

**FIGURE 1:**
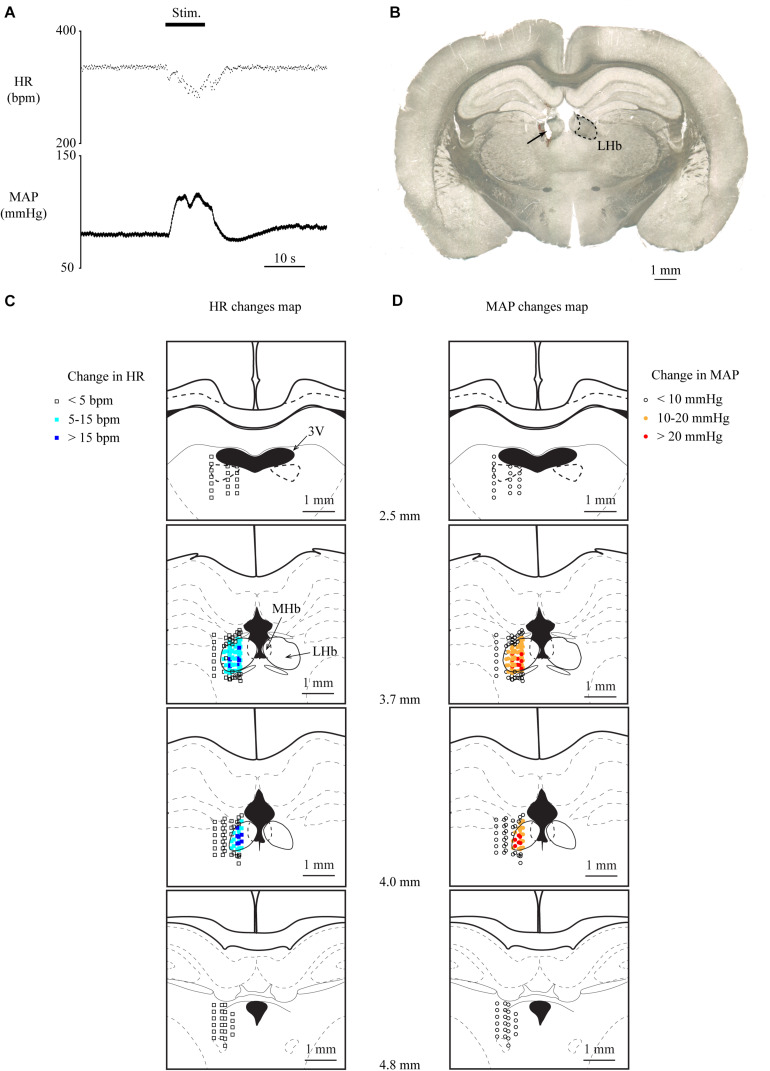
**(A)** Changes in heart rate (HR) and mean arterial pressure (MAP) upon stimulation of the lateral habenula (LHb). The stimulation period (Stim) is indicated by a black bar at the top. **(B)** *Post hoc* confirmation of the stimulation sites. The picture shows a coronal section of the brain 3.7 mm caudal from the bregma. The LHb is surrounded by the dotted line. The arrow indicates the electrically lesioned site made by a stimulation electrode after the recording. **(C,D)** Effective sites of stimulation to the LHb on HR **(C)** and MAP **(D)**. The stimulation sites are illustrated with symbols on the coronal sections that are 2.5–4.8 mm caudal from the bregma. The open squares, sky-blue squares, and blue squares indicate that the response magnitudes of HR were less than 5, 5–15, and greater than 15 bpm, respectively. The open circles, orange circles, and red circles indicate that the response magnitudes of MAP were less than 10, 10–20, and greater than 20 mmHg, respectively. 3V, third ventricle; LHb, lateral habenula; MHb, medial habenula.

To investigate whether the LHb is involved in cardiovascular regulation, we examined the effects of electrical stimulation of the LHb on HR and MAP. Totally, 82 sites inside and 111 sites outside the LHb were stimulated in 13 rats. We commonly observed that stimulation of the LHb decreased HR and increased MAP (Figures 1A,B). On the other hand, stimulation outside of the LHb mostly did not change HR or MAP (Figures 1C,D). When compared with the stimulation sites outside the LHb, a significantly large number of stimulation sites inside the LHb of 12 rats exhibited a decrease of more than 5 bpm in HR [inside, 76/82 sites; outside, 5/111 sites; χ(1) = 150.562, *p* < 0.001, χ^2^ test] and an increase of more than 10 mmHg in MAP [inside the LHb, 76/82 sites; outside, 5/111 sites; χ(1) = 150.562, *p* < 0.001, χ^2^ test]. Moreover, all the outside stimulation sites that induced the change in HR or MAP were adjacent to the border of the LHb. On average, the magnitudes of HR and MAP changes evoked by stimulating the inside of the LHb were significantly larger than those evoked by stimulating the outside [HR: inside, 17.4 ± 11.7 bpm vs. outside, 6.2 ± 1.3 bpm, *t*(63.637) = 7.675, *p* < 0.001, unpaired *t* test; MAP: inside, 22.9 ± 9.2 mmHg vs. outside, 13.8 ± 2.0 mmHg, *t*(20.258) = 6.531, *p* < 0.001, unpaired *t* test].

### Involvement of the Sympathetic and Parasympathetic Nervous Systems in the Cardiovascular Responses to Electrical Stimulation of the LHb

**FIGURE 2:**
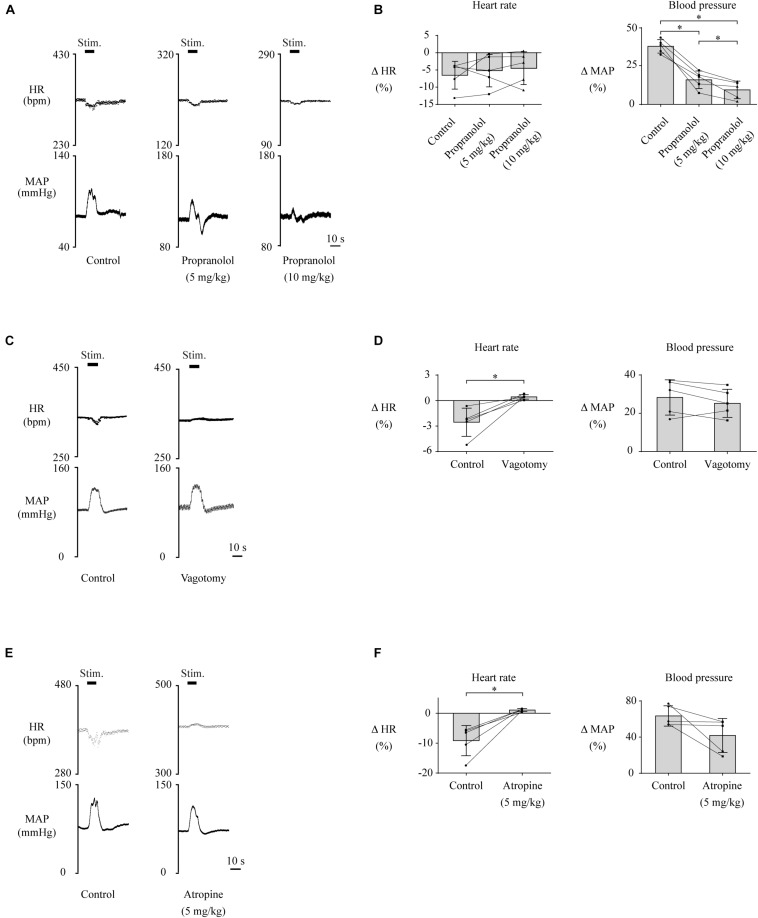
**(A,C,E)** Changes in heart rate (HR) and mean arterial pressure (MAP) upon stimulation of the lateral habenula (LHb) before (control) and after administration of propranolol (5 or 10 mg/kg, i.v.) **(A)**, vagotomy **(C)**, or administration of atropine **(E)**. The stimulation periods (Stim) are indicated by black bars at the top of each chart. **(B,D,F)** Response magnitudes of HR and MAP to stimulations of the LHb before (control) and after administration of propranolol [*n* = 5, **(B)**], vagotomy [*n* = 5, **(D)**], or administration of atropine [*n* = 5, **(F)**]. The asterisks indicate *p* < 0.05. Application of propranolol significantly attenuated the responses of MAP but not of HR. On the other hand, vagotomy and application of atropine significantly suppressed the responses of HR but not of MAP.

Lateral habenula-induced changes in HR and MAP are thought to be mediated by the cardiovascular autonomic nervous system, which consists of sympathetic and parasympathetic nerves. To investigate which nerve is responsible for HR and MAP changes, we pharmacologically blocked the cardiac sympathetic nerve or performed bilateral vagotomy to block the parasympathetic input to the heart and compared the effects of LHb stimulation on HR and MAP across the control, sympathetic blocking, and parasympathetic blocking conditions. The effects of the blockades on baseline HR and MAP are shown in Table 1. Administration of propranolol, a non-selective β-adrenoceptor antagonist that blocks the cardiac sympathetic nerves, significantly attenuated the LHb-induced MAP change as compared with the control condition [control, 38.0 ± 4.4% (31.7 ± 2.7 mmHg); propranolol (5 mg/kg), 16.1 ± 5.8% (18.1 ± 3.2 mmHg); propranolol (10 mg/kg), 9.5 ± 5.8% (10.7 ± 3.1 mmHg); *F*(2, 8) = 82.074, *p* < 0.001, repeated-measures ANOVA; control vs. propranolol (5 mg/kg), *p* = 0.001; control vs. propranolol (10 mg/kg), *p* < 0.001; propranolol (5 mg/kg) vs. propranolol (10 mg/kg), *p* = 0.026, *post hoc* Tukey’s test] but did not significantly influence the LHb-induced HR change [control, −6.5 ± 4.0% (−22.4 ± 6.3 bpm); propranolol (5 mg/kg), −5.2 ± 4.7% (−11.6 ± 4.9 bpm); propranolol (10 mg/kg), −4.5 ± 4.7% (−8.9 ± 4.3 bpm); *F*(2, 8) = 0.562, *p* = 0.591, repeated-measures ANOVA; Figures 2A,B]. In contrast to the blockade of the sympathetic nerves, bilateral vagotomy significantly attenuated the LHb-induced HR change [control, −2.5 ± 1.7% (−8.9 ± 5.4 bpm); vagotomy, 0.4 ± 0.3% (1.5 ± 0.8 bpm); *t*(4) = −3.960, *p* = 0.017, paired *t* test] but did not significantly influence the LHb-induced MAP change [control, 28.3 ± 9.2% (25.5 ± 10.2 mmHg); vagotomy, 25.2 ± 7.3% (25.8 ± 10.3 mmHg); *t*(4) = 1.507, *p* = 0.206, paired *t* test; Figures 2C,D]. Since the vagus nerves include not only cardiac parasympathetic efferents but also a part of afferent fibers from the aortic baroreceptors, we also tested the effects of administration of atropine, a muscarinic receptor antagonist. Administration of atropine suppressed the LHb-induced HR response [control, −9.1 ± 5.0% (−32.8 ± 17.0 bpm); atropine, 1.1 ± 0.5% (4.2 ± 1.9 bpm); *t*(4) = −4.486, *p* = 0.011, paired *t* test] but did not significantly influence the LHb-induced MAP response [control, 63.4 ± 11.2% (44.6 ± 6.1 mmHg); atropine, 41.8 ± 18.8% (26.4 ± 11.2 mmHg); *t*(4) = 2.138, *p* = 0.099, paired *t* test; Figures 2E,F]. Taken together, the LHb seems to regulate MAP and HR *via* the sympathetic and parasympathetic nerves, respectively.

**TABLE 1:** Baseline heart rate (HR) and mean arterial pressure (MAP) before and after vagotomy and blockades of b-adrenergic receptors and muscarinic receptors.

Intervention	HR (bpm)			MAP (mmHg)		
	Control	1st intervention	2nd intervention	Control	1st intervention	2nd intervention
Propranolol	338.8 ± 25.1	219.6 ± 11.7* (5 mg/kg)	194.6 ± 10.5* (10 mg/kg)	82.8 ± 7.0	111.3 ± 6.0* (5 mg/kg)	110.5 ± 9.9* (10 mg/kg)
Vagotomy	354.4 ± 31.8	361.1 ± 24.5	–	88.6 ± 8.7	99.6 ± 14.5	–
Atropine	367.2 ± 35.6	386.8 ± 16.7	–	70.8 ± 5.0	65.2 ± 9.8	–

Values in brackets indicate drug doses. *p < 0.05 vs. control.

### Involvement of the Serotonergic System in the Cardiovascular Responses to Electrical Stimulation of the LHb

**FIGURE 3:**
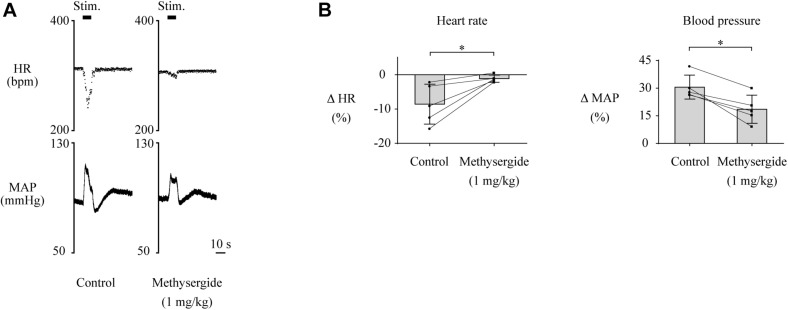
**(A)** Changes in heart rate (HR) and mean arterial pressure (MAP) upon stimulation of the lateral habenula (LHb) before (control) and after administration of a 5-HT receptor antagonist, methysergide (1 mg/kg, i.v.). The stimulation periods (Stim) are indicated by black bars at the top of each chart. **(B)** Response magnitudes of HR and MAP to stimulations of the LHb before (control) and after administration of methysergide (*n* = 5). The asterisks indicate *p* < 0.05. Administration of methysergide significantly suppressed the responses of both HR and MAP.

Although the above results indicate that neuronal signals originating from the LHb affect cardiovascular regulation, the neural circuit that relays the LHb signals to the cardiovascular autonomic system remains to be identified. The serotonergic system, which receives inputs from the LHb and is involved in cardiovascular regulation, could be a major candidate for this neural circuit. To test this idea, we pharmacologically blocked 5-HT receptors and compared the effects of LHb stimulation on HR and MAP. The effects of the blockades on baseline HR and MAP are shown in Table 2. At first, we systematically administered methysergide, a non-selective 5-HT receptor antagonist, and compared the effects of LHb stimulation on HR and MAP across the control and methysergide conditions. Administration of methysergide significantly attenuated both the LHb-induced HR response [control, −8.6 ± 5.8% (−28.4 ± 19.1 bpm); methysergide, −1.1 ± 1.0% (−3.8 ± 3.2 bpm); *t*(4) = −3.311, *p* = 0.030, paired *t* test] and the MAP response [control, 30.6 ± 6.5% (24.5 ± 5.3 mmHg); methysergide, 18.6 ± 7.7% (14.8 ± 6.7 mmHg); *t*(4) = 5.009, *p* = 0.007, paired *t* test; Figures 3A,B].

**TABLE 2:** Baseline heart rate (HR) and mean arterial pressure (MAP) before and after blockades of 5-HT receptors.

Intervention	HR (bpm)			MAP (mmHg)		
	Control	1st intervention	2nd intervention	Control	1st intervention	2nd intervention
Methysergide	329.2 ± 27.4	318.5 ± 32.2*	–	80.8 ± 11.7	77.0 ± 21.4	–
Mianserin	334.0 ± 24.7	323.7 ± 17.8	–	77.8 ± 9.7	54.1 ± 10.2*	–
NAD-299	324.2 ± 18.2	300.9 ± 10.4* (0.3 mg/kg)	284.0 ± 9.7* (0.9 mg/kg)	80.6 ± 4.9	80.0 ± 19.6 (0.3 mg/kg)	62.9 ± 21.2 (0.9 mg/kg)
GR-127935	317.2 ± 30.5	295.8 ± 31.0* (3 mg/kg)	284.2 ± 28.7* (6 mg/kg)	92.7 ± 8.4	96.4 ± 9.2 (3 mg/kg)	97.3 ± 8.1 (6 mg/kg)
SB-269970	335.1 ± 12.1	336.6 ± 13.8 (1 mg/kg)	335.6 ± 26.2 (2 mg/kg)	87.1 ± 7.5	85.8 ± 15.6 (1 mg/kg)	80.3 ± 19.7 (2 mg/kg)

Values in brackets indicate drug doses. *p < 0.05 vs. control.

**FIGURE 4:**
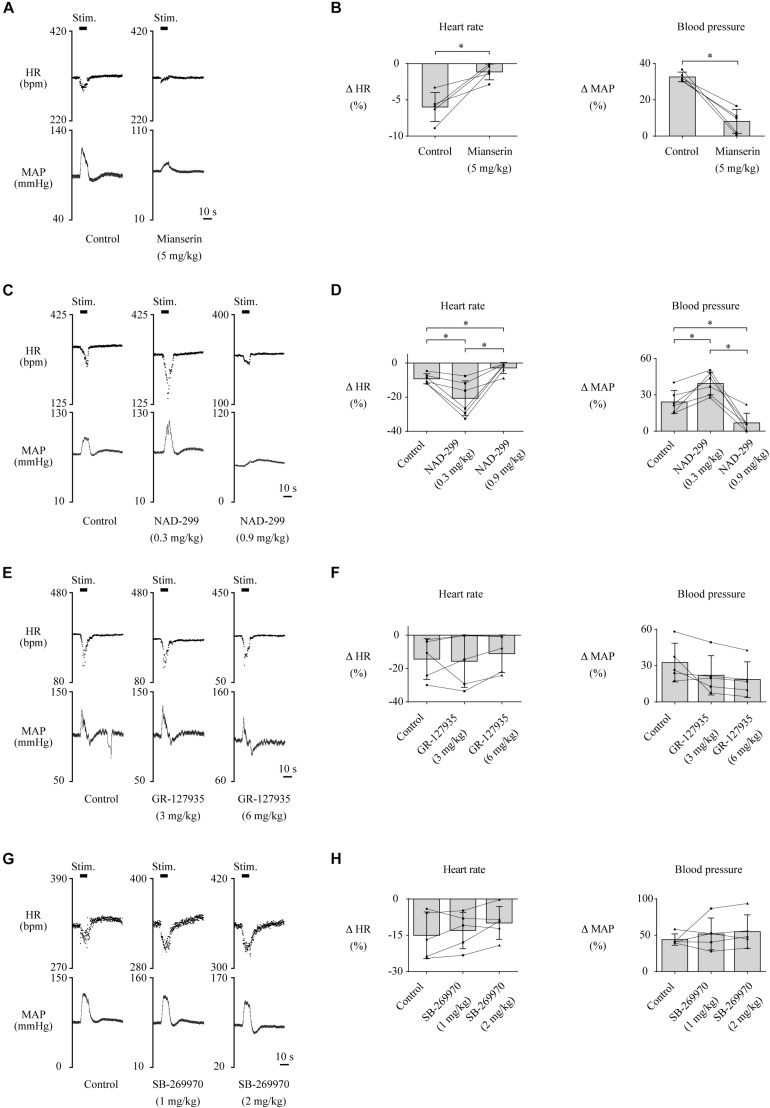
**(A,C,E,G)** Changes in heart rate (HR) and mean arterial pressure (MAP) upon stimulation of the lateral habenula (LHb) before (control) and after administration of a 5-HT_2_ receptor antagonist, mianserin (5 mg/kg, i.v.) **(A)**; a 5-HT_1A_ receptor antagonist, NAD-299 (0.3 or 0.9 mg/kg, i.v.) **(C)**; a 5-HT_1B/1D_ receptor antagonist, GR-127935 (3 or 6 mg/kg, i.v.) **(E)**; and a 5-HT_7_ receptor antagonist, SB-269970 (1 or 2 mg/kg, i.v.) **(G)**. The stimulation periods (Stim) are indicated by black bars at the top of each chart. **(B,D,F,H)** Response magnitudes of HR and MAP to stimulations of the LHb before (control) and after administration of mianserin [*n* = 5, **(B)**], NAD-299 [*n* = 6, **(D)**], GR-127935 [*n* = 5, **(F)**], and SB-269970 [*n* = 5, **(H)**]. The asterisks indicate *p* < 0.05. The presence of a low dose of NAD-299 enhanced the responses of both HR and MAP, but that of a high dose of NAD-299 or mianserin attenuated them.

Furthermore, we examined which subtypes of the 5-HT receptor are involved in the cardiovascular responses. Administration of mianserin, a 5-HT_2_ receptor antagonist, significantly attenuated both the LHb-induced HR response [control, −6.0 ± 2.0% (−20.2 ± 7.2 bpm); mianserin, −1.1 ± 1.1% (−3.8 ± 3.8 bpm); *t*(4) = −4.413, *p* = 0.012, paired *t* test] and the MAP response [control, 32.7 ± 2.6% (25.2 ± 1.5 mmHg); mianserin, 8.1 ± 6.6% (4.9 ± 4.1 mmHg); *t*(4) = 6.289, *p* = 0.003, paired *t* test; Figures 4A,B]. Administration of NAD-299, a 5-HT_1A_ receptor antagonist, had dose-dependent effects on the LHb-induced HR response [control, −9.2 ± 3.0% (−29.7 ± 3.6 bpm); NAD-299 (0.3 mg/kg), −20.7 ± 10.2% (−61.9 ± 12.2 bpm); NAD-299 (0.9 mg/kg), −2.8 ± 3.2% (−8.1 ± 3.9 bpm); *F*(1.048, 5.239) = 15.972, *p* = 0.009, repeated-measures ANOVA] and MAP response [control, 24.1 ± 9.6% (19.5 ± 3.3 mmHg); NAD-299 (0.3 mg/kg), 39.4 ± 9.5% (33.5 ± 3.1 mmHg); NAD-299 (0.9 mg/kg), 8.9 ± 8.8% (6.0 ± 2.9 mmHg); *F*(2, 10) = 26.689, *p* < 0.001, repeated-measures ANOVA; Figures 4C,D]; that is, the application of a low dose of the drug significantly enhanced the LHb-induced HR response (*p* = 0.018 vs. control, *post hoc* Tukey’s test) and that of a high dose significantly attenuated the response (*p* = 0.004 vs. control, *post hoc* Tukey’s test). Moreover, application of the low dose significantly enhanced the LHb-induced MAP response (*p* = 0.011 vs. control, *post hoc* Tukey’s test) and that of the high dose significantly attenuated the response (*p* = 0.006 vs. control, *post hoc* Tukey’s test). Meanwhile, application of GR-127935, a 5-HT_1B_ and 5-HT_1D_ receptor antagonist, had no significant effect on the LHb-induced HR response [control, −14.4 ± 12.2% (−43.9 ± 16.4 bpm); GR-127935 (3 mg/kg), −15.7 ± 15.7% (−42.7 ± 18.6 bpm); GR-127935 (6 mg/kg), −11.1 ± 11.3% (−29.3 ± 12.8 bpm); *F*(2, 8) = 0.617, *p* = 0.563, repeated-measures ANOVA] or the MAP response [control, 32.6 ± 6.0% (29.4 ± 5.6 mmHg); GR-127935 (3 mg/kg), 22.1 ± 16.2% (21.6 ± 7.1 mmHg); GR-127935 (6 mg/kg), 18.5 ± 14.7% (18.4 ± 6.6 mmHg); *F*(1.029, 4.116) = 5.058, *p* = 0.086, repeated-measures ANOVA; Figures 4E,F]. In addition, administration of SB-269970, a 5-HT_7_ receptor antagonist, had no significant effect on the LHb-induced HR response [control, −15.0 ± 9.6% (−50.8 ± 14.8 bpm); SB-269970 (1 mg/kg), −13.0 ± 7.5% (−44.3 ± 11.5 bpm); SB-269970 (2 mg/kg), −9.9 ± 6.8% (−33.0 ± 10.2 bpm); *F*(2, 8) = 2.567, *p* = 0.138, repeated-measures ANOVA] or the MAP response [control, 44.0 ± 7.8% (37.9 ± 1.7 mmHg); SB-269970 (1 mg/kg), 51.8 ± 22.0% (41.8 ± 4.2 mmHg); SB-269970 (2 mg/kg), 55.0 ± 23.1% (40.6 ± 2.6 mmHg); *F*(1.068, 4.272) = 0.788, *p* = 0.431, repeated-measures ANOVA; Figures 4G,H]. These results suggest that the LHb regulates HR and MAP at least partly through the serotonergic system, particularly *via* the 5-HT_1A_ and 5-HT_2_ receptors but not the 5-HT_1B_, 5-HT_1D_, or 5-HT_7_ receptor.

## Discussion

In anesthetized rats, electrical stimulation of the LHb caused a decrease in HR and an increase in MAP. The LHb-induced HR response was attenuated by either vagotomy or blockade of muscarinic receptors. The LHb-induced MAP response was also attenuated by blockade of β-adrenoceptors. Furthermore, the LHb-induced HR and MAP responses were attenuated by administration of a non-selective 5-HT receptor antagonist. In particular, these responses were attenuated by a 5-HT_2_ receptor antagonist or a high dose of a 5-HT_1A_ receptor antagonist. Our findings suggest that activation of the LHb causes cardiovascular responses *via* excitation of the cardiovascular sympathetic and cardiac parasympathetic nervous systems and that these responses are mediated at least partly by the central serotonergic system *via* the 5-HT_1A_ and 5-HT_2_ receptors.

Recently, pharmacological disinhibition of the LHb with microinjections of bicuculline, a GABA_*A*_ receptor antagonist, has been used to evoke thermal responses with cardiovascular responses (Ootsuka and Mohammed, 2015; Ootsuka et al., 2017). However, the pharmacological disinhibition evokes slow and long-lasting responses because the microinjections cause gradual and continuous disinhibition of GABAergic inputs to the LHb neurons. On the other hand, to stress events, only phasically activated neurons in the LHb have been reported, but continuously responding neurons have not been done yet (Gao et al., 1996; Matsumoto and Hikosaka, 2009; Kawai et al., 2015; Li et al., 2019). Moreover, although electrical activation of the unilateral LHb is enough to modulate behavioral responses (Matsumoto and Hikosaka, 2011), unilateral pharmacological disinhibition by bicuculline looks not enough to induce cardiovascular and thermal responses in the previous reports. Therefore, we electrically activated the LHb neurons to mimic fast and short activation to stress events rather than pharmacological disinhibition. Actually, in response to the electrical stimulation of the LHb, HR and MAP reached maximal responses in a few seconds.

Although we observed that electrical stimulation of the LHb induced bradycardia, a previous study reported tachycardia evoked by such stimulation (Ootsuka and Mohammed, 2015). Please note that we observed a decrease in HR, as well as an increase in MAP, when stimulating only the insides of the LHb, whereas a previous study reported increases in HR and MAP when the inside of the LHb was stimulated and there were changes in them when even the outside of the LHb was stimulated. Therefore, stimulations in our study had higher spatial specificity than those in the previous report. This difference of spatial specificity might be accounted for by the difference in the stimulation intensity and the electrode between these studies. In the previous report, they used a higher stimulation intensity (0.5–1 mA) than that used in our study (300 mA). They also stimulated the LHb with a monopolar electrode, but we did with a coaxial electrode. Therefore, the specific activation of the LHb might cause bradycardia. On the other hand, even when we tested to stimulate the LHb with a higher stimulation intensity (1 mA) or the same stimulation parameters with the previous report, we could observe only bradycardia but not tachycardia (Supplementary Figure S1). Although we could not completely reproduce the stimulation which was used in the previous report because of using a different type of electrode, the discrepancy in the LHb stimulation-induced HR responses might be also caused by other reasons—the difference in the anesthesia (a mixture of urethane and α-chloralose vs. urethane) or the strain (Sprague–Dawley vs. Wistar).

Notably, the cardiovascular responses evoked by the LHb stimulation are similar to those observed in animals showing “freezing” rather than “fight or flight” as a behavioral defense response to stressful events. It has been known that stressful events cause either freezing or fight-or-flight behavior (Hilton, 1982; Schenberg et al., 2005). In freezing animals, bradycardia and elevation of systemic arterial pressure are commonly observed, whereas in fight-or-flight animals, tachycardia and an increase in systemic arterial pressure are seen (Hilton, 1982; Schenberg et al., 1993; Schadt and Hasser, 1998; Carrive, 2006; Wang et al., 2010). Therefore, activation of the LHb might mimic the brain state that induces freezing.

Cardiovascular activities are controlled by the sympathetic and parasympathetic systems. Our study investigated the mechanism by which the LHb affects these systems to regulate cardiovascular functions. With the administration of a β-adrenoceptor antagonist, the LHb-induced MAP response was partly suppressed, whereas the LHb-induced HR response was not significantly affected. This indicates that the pressor response was mediated at least partly by an increase in cardiac output with activation of the cardiac sympathetic nerves *via* the b_1_-adrenoceptor. Moreover, since the blockade of the β-adrenoceptor did not completely eliminate the LHb-induced MAP response even at the high dose, total peripheral vascular resistance might be increased by stimulation *via* excitation of the sympathetic vasoconstrictor fibers. As a thermal regulatory response, pharmacological disinhibition of the LHb also causes excitation of the sympathetic nerves that innervate brown adipose tissue (Ootsuka and Mohammed, 2015; Ootsuka et al., 2017). In the freezing behavior mentioned above, activation of the cardiac sympathetic nerves and increases in total peripheral vascular resistance that are induced by excitation of the sympathetic vasoconstrictor fibers other than those of the muscle vasculature are also reported in conscious rats (Carrive, 2006; Yoshimoto et al., 2010). Freezing behavior has been recognized as a preparatory response for the active coping strategy, such as the fight-or-flight response (Miki and Yoshimoto, 2010). Thus, the LHb-induced sympathetic changes contribute to redistribution of blood flow mainly to the skeletal muscles, which strongly increases the metabolic rate in the active behavior followed by the freezing behavior. In the freezing behavior of conscious rats, simultaneous excitations of the cardiac sympathetic and parasympathetic nerves are induced, and bradycardia is observed (Carrive, 2006). In our study, bilateral vagotomy and the blockade of muscarinic receptors tended to change the LHb-induced HR response from bradycardia to tachycardia. Moreover, a part of the LHb-induced MAP response was mediated by β-adrenoreceptor activation. Therefore, the balance between the effects of the excitations of the cardiac sympathetic and parasympathetic nerves determined the HR response, bradycardia. Freezing-related bradycardia may have a role in reducing excessive elevation of systemic arterial pressure, which is caused by sympathoexcitation (Miki and Yoshimoto, 2010).

It has been reported that neurons in the LHb respond to stressful events and regulate behavioral responses to these events *via* the monoaminergic systems (Hikosaka, 2010). Among the monoaminergic systems, the central serotonergic system including the dorsal and the median raphé nuclei is known not only to mediate stress-induced behavioral responses but also to play crucial roles in cardiovascular autonomic regulation (Smits et al., 1978; Kuhn et al., 1980; Alvarenga et al., 2005), which includes cardiovascular responses to stressful events (Nalivaiko and Sgoifo, 2009; Ikoma et al., 2018). In the present study, we observed that pharmacologic blockade of 5-HT receptors attenuated both the bradycardia and the pressor response induced by LHb stimulation, suggesting that the pathway from the LHb to the serotonergic system regulates these cardiovascular responses. Since we administered the 5-HT receptor blocker, methysergide, intravenously, it is difficult to determine precisely which part of the serotonergic system is involved in this regulation (e.g., the central vs. the peripheral serotonergic system). However, the concentration of serotonin in plasma is very low and its peripheral vascular effect only takes place after release from platelets (Nalivaiko and Sgoifo, 2009). Therefore, our results indicate that the central serotonergic system, rather than the peripheral one, mediates the LHb-induced cardiovascular responses. Methysergide not only has antagonistic effects to 5-HT receptors but also has partial agonistic effects to the receptors (Colpaert et al., 1979; Terrón, 1997). Therefore, we also tried to examine the effects of administering selective antagonists of the 5-HT receptor subtypes on the LHb-induced HR and MAP responses.

The role of the serotonergic system in cardiovascular regulation depends on 5-HT receptor subtypes (Ramage, 2001). It has been reported that activation of 5-HT_1A_ receptors attenuates stress-induced changes in HR, systemic arterial pressure, and behavioral responses (Ngampramuan et al., 2008). Moreover, in 5-HT_1A_ KO mice, cardiovascular and behavioral responses to stressful events are enhanced as compared with those in WT mice (Gross et al., 2000; Pattij et al., 2002). Thus, in the serotonergic system, 5-HT_1A_ receptors seem to suppressively modulate the stress-related activity of the cardiovascular autonomic nervous system. Consistent with this literature, we observed that administration of a low dose (0.3 mg/kg, i.v.) of a 5-HT_1A_ receptor antagonist, NAD-299, enhanced the LHb-induced HR and MAP responses. NAD-299 is more selective to 5-HT_1A_ receptors than other 5-HT_1A_ receptor antagonists with a low affinity for adrenergic, dopaminergic, and muscarinic receptors especially in the dose which we used in this study (Johansson et al., 1997; Madjid et al., 2006). Although the neural circuit underlying the 5-HT_1A_ receptor-mediated cardiovascular regulation is not immediately clear, a previous study reported that administration of a 5-HT_1A_ receptor antagonist into the medullary raphé nucleus attenuates the response of the HR to stressful events but does not affect the response of the systemic arterial pressure to the events, probably owing to activation of 5-HT_1A_ autoreceptors (Nalivaiko et al., 2005). Therefore, the medullary raphé nucleus is likely to be a part of the neural circuit underlying the 5-HT_1A_ receptor-mediated cardiovascular regulation.

In contrast to the low-dose administration, that of the high dose (0.9 mg/kg, i.v.) of the 5-HT_1A_ receptor antagonist attenuated the LHb-induced HR and MAP responses. Similar heterogeneous effects on cardiovascular regulation have been reported (Ramage, 2001). For instance, administration of 5-HT_1A_ receptor agonists at low doses causes an increase in blood pressure and sympathoexcitation, but that at high doses causes a decrease in blood pressure and sympathoinhibition (Dedeoğlu and Fisher, 1991; Anderson et al., 1992). Such heterogeneous effects also occur depending on the administration region. That is, administration of 5-HT_1A_ receptor agonists into the raphé obscurus induces pressor responses (Dreteler et al., 1991), but that into the dorsal raphé, raphé magnus, and pallidus induces depressor responses (McCall and Clement, 1994). Therefore, the dose-dependent effects of 5-HT_1A_ receptor agonists and antagonists might be caused by different functions and affinities of endogenous serotonin and drugs to 5-HT_1A_ receptors depending on the regions in the brain. We further tested the effect of administrations of 5-HT_1B_ and 5-HT_1D_ receptor antagonists, but they did not affect the LHb-induced HR and MAP responses. In addition to the antagonists for 5-HT_1A_, 5-HT_1B_, and 5-HT_1D_ receptors, we also administered a non-selective 5-HT_2_ receptor antagonist and observed that it attenuated the LHb-induced HR and MAP responses. Consistent with our observation, previous studies have shown that administration of 5-HT_2_ receptor agonists, especially for 5-HT_2A_ receptors, causes sympathoexcitatory effects (McCall and Clement, 1994; Ramage, 2001; Nalivaiko and Sgoifo, 2009). It has been reported that mianserin, which was used as a 5-HT_2_ receptor antagonist in this study, also blocks α_1_ and α_2_ adrenoreceptors and inhibits noradrenaline reuptake (Cavero et al., 1980, 1981). In this study, we cannot ignore the non-specific effects to the LHb-induced responses. However, the non-specific effects probably did not induce the attenuation of the LHb-induced responses at least at the peripheral level because the effects of both blocking presynaptic α_2_ adrenoreceptors and inhibiting noradrenaline reuptake elevate the noradrenaline level, and noradrenaline-induced HR and MAP increases are not changed in pithed rats (Cavero et al., 1980). Administration of a 5-HT_7_ antagonist did not affect the LHb-induced changes in HR and arterial pressure. Taken together, our findings suggest that 5-HT_1A_ and 5-HT_2_ receptors selectively participate in the neural circuit including the LHb and central serotonergic system to mediate cardiovascular regulation.

Hereafter, the LHb and the serotonergic system-involved neural circuit for cardiovascular regulation need to be examined in detail. Moreover, since we used anesthetized rats in this study, the use of conscious animals may help to understand the neural mechanism of the LHb-regulating autonomic cardiovascular responses while observing stress-induced behavioral responses.

## Data Availability Statement

The raw data supporting the conclusions of this article will be made available by the authors, without undue reservation.

## Ethics Statement

The animal study was reviewed and approved by the Animal Experimental Committee of the University of Tsukuba.

## Author Contributions

TD conducted the experiment, analyzed the data, and wrote the manuscript. YS conducted the experiment and reviewed the manuscript. MM and TK conceived the study, acquired funding, and reviewed the manuscript. All authors contributed to the article and approved the submitted version.

## Conflict of Interest

The authors declare that the research was conducted in the absence of any commercial or financial relationships that could be construed as a potential conflict of interest.

## Footnotes

1 http://www.jslc.co.jp/english/animals/rat.php

## Abbreviations

3V: third ventricle
5-HT: serotonin
HR: heart rate
i.p.: intraperitoneal
i.v.: intravenous
LHb: lateral habenula
MAP: mean arterial pressure
MHb: medial habenula
Stim: stimulation.
