# Supplementary material for: Lateral Habenula Regulates Cardiovascular Autonomic Responses via the Serotonergic System in Rats
Source: Front Neurosci. 2021 Mar 29;15:655617. doi: 10.3389/fnins.2021.655617 (PMC8039147; doi:10.3389/fnins.2021.655617)
Supplement: Supplementary file 1 [file Image_1.PDF]

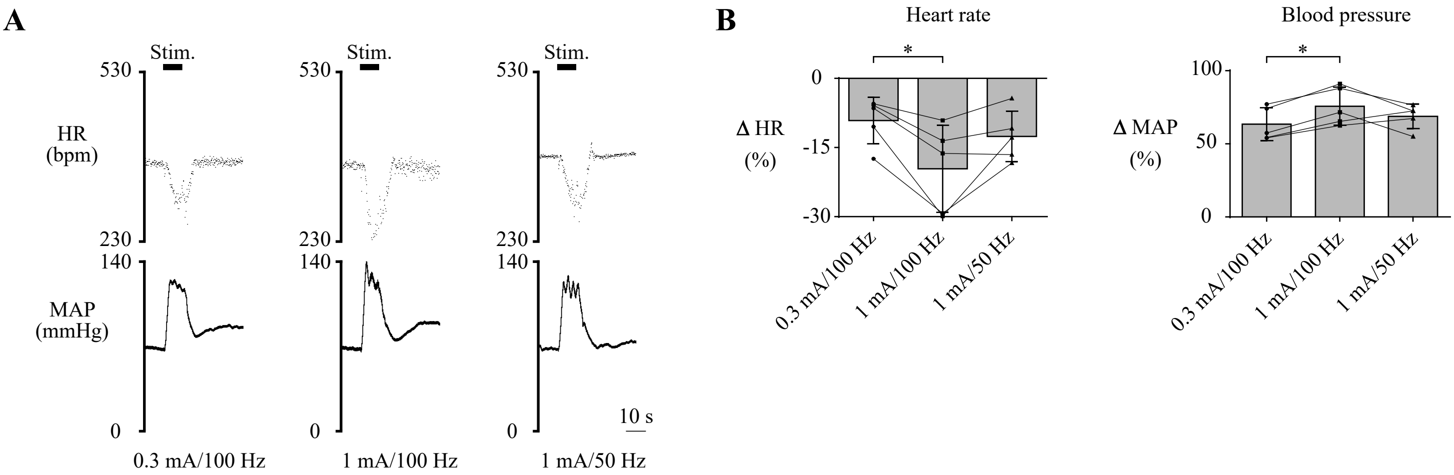

Supplementary Figure S1. A, Changes in heart rate (HR) and mean arterial pressure (MAP) upon stimulation of the lateral habenula (LHb) with 300 µA intensity, 0.5 ms pulse duration and 100 Hz frequency (300 µA/100 Hz), 1 mA intensity, 0.5 ms pulse duration and 100 Hz frequency (1 mA/100 Hz), and 1 mA intensity, 1 ms pulse duration, 50 Hz frequency (1 mA/50 Hz). The stimulation periods (Stim.) are indicated by black bars at the top of each chart. **B,** Response magnitudes of HR and MAP to stimulations of the LHb with 300 µA, 0.5 ms pulse duration, 100 Hz frequency (300 µA/100 Hz), 1 mA intensity, 0.5 ms pulse duration and 100 Hz frequency (1 mA/100 Hz) and 1 mA intensity, 1 ms pulse duration and 50 Hz frequency (1 mA/50 Hz). The asterisks indicate $p < 0.05$. The stimulations of the LHb always evoked a decrease in HR and an increase in MAP (n = 5).
